# Review of Vision-Based Deep Learning Parking Slot Detection on Surround View Images

**DOI:** 10.3390/s23156869

**Published:** 2023-08-02

**Authors:** Guan Sheng Wong, Kah Ong Michael Goh, Connie Tee, Aznul Qalid Md. Sabri

**Affiliations:** 1 Faculty of Information Science and Technology, Multimedia University, Melaka 75450, Malaysia; tee.connie@mmu.edu.my; 2 Faculty of Computer Science and Information Technology, University of Malaya, Kuala Lumpur 50603, Malaysia; aznulqalid@um.edu.my

**Keywords:** deep learning, parking slot detection, surround view images

## Abstract

Autonomous vehicles are gaining popularity, and the development of automatic parking systems is a fundamental requirement. Detecting the parking slots accurately is the first step towards achieving an automatic parking system. However, modern parking slots present various challenges for detection task due to their different shapes, colors, functionalities, and the influence of factors like lighting and obstacles. In this comprehensive review paper, we explore the realm of vision-based deep learning methods for parking slot detection. We categorize these methods into four main categories: object detection, image segmentation, regression, and graph neural network, and provide detailed explanations and insights into the unique features and strengths of each category. Additionally, we analyze the performance of these methods using three widely used datasets: the Tongji Parking-slot Dataset 2.0 (ps 2.0), Sejong National University (SNU) dataset, and panoramic surround view (PSV) dataset, which have played a crucial role in assessing advancements in parking slot detection. Finally, we summarize the findings of each method and outline future research directions in this field.

## 1. Introduction

In recent years, the development of autonomous vehicles has undergone rapid growth, and automated parking systems have emerged as key components in this technological advancement. A critical aspect of ensuring the reliability and effectiveness of automated parking systems lies in the accurate detection of parking slots. Traditionally, sensors such as ultrasonic or infrared sensors have been employed to gather information about the surrounding parking space. However, these traditional sensors often fall short in providing a comprehensive and precise representation of the parking environment. Conversely, vision-based parking slot detection has demonstrated promising results, particularly when utilizing deep learning approaches. Such methods offer enhanced robustness and accuracy compared to alternative techniques.

Vision-based deep learning approaches have revolutionized various computer vision tasks, including object detection [1,2] and image segmentation [3]. They could also extend their application in the field of autonomous driving, where accurate perceptions of the surround view are crucial. Parking slot detection using surround view images has gained significant attention due to its ability to provide a holistic view of the parking scene, combining information from multiple camera perspectives.

In this review paper, our primary focus will be on vision-based deep learning techniques for parking slot detection, specifically targeting the precise detection of a parking slot’s location for autonomous vehicles, rather than parking slot occupancy detection for parking management systems. Our aim is to thoroughly analyze and evaluate the different methodologies and architectures employed in this field. By providing an overview of the existing literature, this review aims to highlight the progress made, challenges faced, and potential future directions in the field.

**Figure 1:**
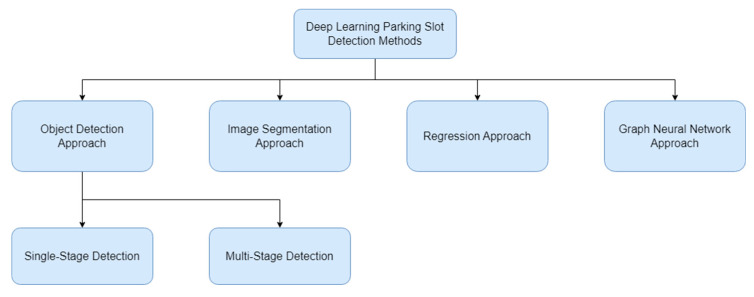
Deep-learning-based parking slot detection methods.

To achieve our goal, we will categorize the parking slot detection techniques into several distinct categories. These categories include Object Detection-Based approaches [4,5,6,7,8,9,10,11,12,13,14,15,16,17,18], which employ either Single-stage Detection or Multi-stage Detection techniques to locate the parking slot in the image. We will also explore Image Segmentation-Based techniques [19,20,21,22,23,24,25,26], which involve segmenting the parking slots lines from the background to achieve accurate detection. Additionally, we will investigate Regression-Based methods [8,27,28] that predict the position of the marking points/corner of a parking slot. Lastly, there is the approach using Graph Neural Networks [29], which realizes the relationship between the marking points. The categorization method is shown in Figure 1.

Furthermore, we provide a comprehensive analysis of the benchmark datasets commonly used for training and evaluating parking slot detection algorithms, the namely ps 2.0 dataset [4], SNU dataset [6], and PSV dataset [19]. We discuss the challenges associated with these datasets, including variations in lighting conditions, occlusions, and different types of parking slots.

Finally, we summarize the findings of our review and highlight the current trends, limitations, and future research directions for parking slot detection on surround view images. By presenting an extensive overview of the existing literature, this review paper aims to provide a valuable resource for researchers.

In Section 2, we will discuss the motivation behind this work and outline the research procedure that was undertaken to systematically select relevant studies for review. Section 3 will delve into the various deep learning methods used for parking slot detection. In Section 4, we will provide detailed information on the current benchmark dataset used specifically for surround view parking slot detection. Finally, in Section 5, we will summarize our review and propose future research directions that can further advance the field of vision-based parking slot detection.

## 2. Research Procedure

### 2.1. Motivation

Parking slot detection on surround view images is a rapidly growing research field that has gained significant attention in recent years. Various methods, ranging from traditional computer vision approaches [30], machine learning approaches [31], to deep learning approaches [6], have been employed to tackle this task However, there is a scarcity of review papers specifically focusing on this topic. Currently, the only existing review paper is by Ma et al. [32] and provides a comprehensive overview of parking slot detection methods, including free-based, parking-marking-based, user-interface-based, and infrastructure-based approaches.

In the subsequent year, Ma et al. [33] published another review paper that specifically focuses on vision-based methods, categorizing them into traditional-based and deep-learning-based approaches. Although this review paper provides valuable insights, the ever-evolving field of deep learning warrants a more detailed and in-depth analysis of the techniques and approaches utilized for parking slot detection.

In addition, Suhr and Jung [34] have also published a similar review paper that categorizes automatic parking systems into user-interface-based, free-space-based, parking-slot-marking-based, and intelligent parking management system (IPMS)-based approaches. Their paper offers valuable insights into two publicly available datasets: the ps 2.0 [4] and SNU dataset [6]. The advantages and disadvantages of these datasets are meticulously tabulated, providing a comprehensive analysis for researchers in the field.

Motivated by the current research trends and the need for a comprehensive review of deep-learning-based parking slot detection methods, we have embarked on writing this review paper. Our aim is to delve into the various deep learning techniques, architectures, and algorithms employed in the context of parking slot detection on surround view images. By synthesizing and analyzing the existing literature, we seek to provide a valuable resource for researchers and practitioners in the field of autonomous vehicles and parking slot detection.

### 2.2. Research Question

To establish a clear guideline and define the scope and purpose of the review, several research questions were formulated:

1. How many types of deep learning approaches are utilized in parking slot detection?
2. What are the specific techniques and methodologies employed to detect parking slots using deep learning?
3. What are the strengths and limitations associated with each deep learning method used for parking slot detection?
4. Which benchmark datasets have been commonly employed to evaluate the performance of these methods?
5. What are the evaluation metrics employed to assess the accuracy and effectiveness of the deep-learning-based parking slot detection methods?

Questions 1–3 are addressed in Section 3, and questions 4–5 are addressed in Section 4.

### 2.3. Research Inclusion/Exclusion Criteria

To ensure that we obtained the relevant works for our review paper, we established inclusion and exclusion criteria to guide our search process. The inclusion criteria were designed to focus on works specifically related to parking slot detection using surround view images. The criteria were as follows:

- Inclusion Criteria (IC)
  ○ IC_1_: Include works that address the topic of parking slot detection.
  ○ IC_2_: Include works that utilize surround view images.

Using these inclusion criteria, we conducted a thorough search across various digital resources, including arXiv, Google Scholar, IEEE Explorer, and MDPI. This initial search yielded a total of 87 works.

To further refine our selection and narrow down the works that align with our objectives, we implemented a set of exclusion criteria. The exclusion criteria were as follows:

- Exclusion Criteria (IC)
  ○ EC_1_: Exclude works that are not written in English.
  ○ EC_2_: Exclude works that utilize surveillance cameras or are not relevant to the view of autonomous vehicles.
  ○ EC_3_: Exclude works that do not employ deep learning approaches.

By applying these exclusion criteria, we carefully evaluated each work and filtered out those that did not meet our requirements. As a result, we arrived at a finalized selection of 26 works that met all the specified criteria.

**Figure 2:**
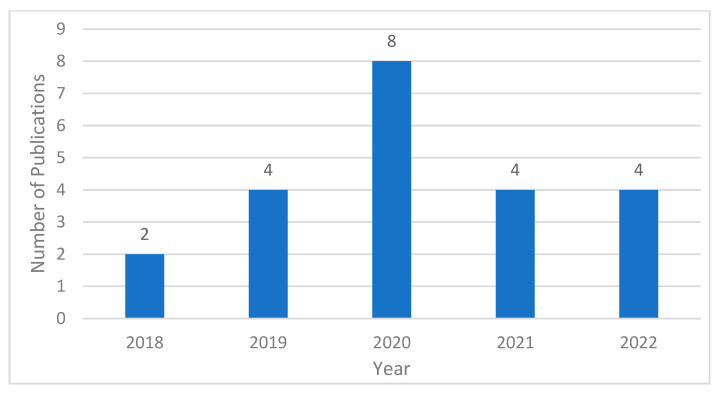
Number of publications by year.

The details of these 26 works, including their publication year, publisher, and main approaches, are presented in Table 1 provided below. Figure 2 shows the graph of number of publications against years. These works will serve as the foundation for our comprehensive review of vision-based deep learning parking slot detection on surround view images.

### 2.4. Contributions of This Work

We present a comprehensive review of deep learning vision-based parking slot detection techniques, encompassing the following key features:

- Diligent Review and Tabulation: To the best of our knowledge, we have meticulously reviewed nearly all published papers on deep-learning-based parking slot detection methods to date. Our thorough analysis has resulted in the meticulous tabulation of the obtained results, making a valuable contribution to the research community.
- First Deep-Learning-Centered Survey: This survey serves as the pioneering deep-learning-centered exploration of parking slot detection. It aims to provide valuable insights and guidance to researchers interested in applying deep learning techniques to their future parking slot detection research endeavors.
- Categorization of Methodologies: The survey categorizes the techniques based on their primary methodologies, which include object detection, image segmentation, regression, and graph neural networks. Notably, this study is the first systematic survey in the domain that includes graph neural networks for parking slot detection. This comprehensive review paper facilitates the comparison and evaluation of these methods.
- Addressing Research Gaps and Guiding Future Exploration: Our paper not only presents an overview of existing literature but also identifies research gaps in the field of parking slot detection. It offers valuable directions for future researchers to identify and explore these gaps, thereby contributing to the advancement of the domain.

## 3. Deep Learning Parking Slot Detection Methods

In this section, we will explore various deep learning methods employed for parking slot detection. Deep learning has revolutionized the field of computer vision, offering powerful tools to tackle complex detection tasks. We will delve into different approaches utilized in the context of parking slot detection, including object detection, image segmentation, regression, and Graph Neural Networks (GNN). A total amount of 26 studies have been carefully reviewed and the findings and insights gathered from these studies will be presented in this section. Each approach will be thoroughly analyzed, highlighting their key components, algorithms, and performance metrics.

### 3.1. Object Detection Approach

Most of the object detection methods utilize the parking slot marking point as their primary detection object as it provides rich information about the parking slot position and orientation. Although marking points could provide a lot of information and further inference to determine the parking slot location, some of the methods use a region proposal network to find the region of interest (ROI) of a parking slot in an image and further deduce the parking slot location. We will further categorize these methods into single-stage detection and multi-stage detection. Single-stage detection methods typically prioritize fast detection speed, often sacrificing a bit of accuracy. On the other hand, multi-stage detection techniques can provide higher accuracy at the cost of slightly slower processing speed.

#### 3.1.1. Single-Stage Detection

Defining single-stage detection in the context of parking slot detection can be ambiguous. Although methods like You Only Look Once (Yolo) [35] are often considered single-stage detectors, they may still incorporate multiple stages or post-processing to accurately identify parking slots. To provide clearer definition, we categorize methods as single-stage detection only if they can identify parking slot candidates directly from a single-stage neural network output, without relying on additional template matching, classification, or image processing. This classification emphasizes the ability to produce desired detection results in a single pass, without further computational steps.

**Figure 3:**
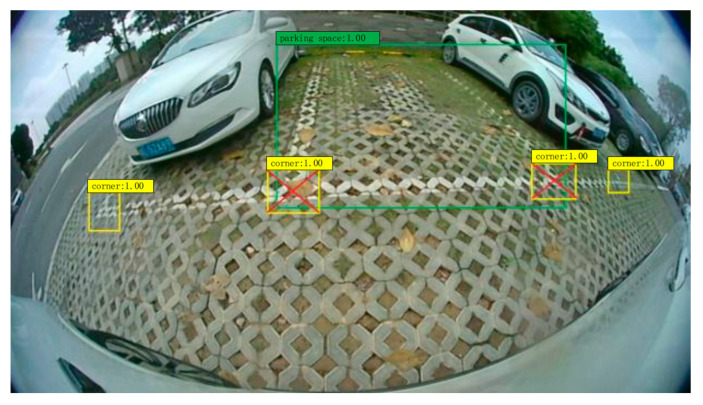
Detection result of Xu and Hu method [9], green box indicates the parking slot and yellow box indicates the parking slot corners.

Xu and Hu [9] took a distinct approach by conducting parking slot detection on fish-eye images instead of panoramic view images, as shown in Figure 3, due to the impact of splicing accuracy and cumulative errors. They modified and pruned the Yolo v3 into 25% of its original size to achieve real time detection speed. Their method included two objects to be detected: one focused on detecting the entire parking slot (1), while the other specifically identified the parking slot corners (2). The empty parking slot would be determined when there were more than two parking slot corners (2) and distance between the corners was sufficient to form an entrance and were located inside the parking slot space (1).

Xie and Wei [11] have applied a soft attention mechanism on the Yolo v3 model and added on six modifications on the original Yolo v3 training process.

1. The Yolo v3 input resolution has been reduced to 288 × 288 to detect the parking slot or car in the images. Due to the size of parking slots and cars being relatively large in the image, reducing the input resolution could help improve the detecting of the parking slot.
2. The momentum value in the optimizer has been increased to 0.92 to have better stability and avoid local minima.
3. The weight decay or regularization parameter has been increased to 0.0015 from 0.0005 to handle overfit during the training process.
4. The batch size has been increased to an optimal value of 160 to have a better convergence during the gradient descent, yet it does not consume much computation memory.
5. The data augmentation crop value has been set at 0.2 to increase the number of data and reduce overfitting.
6. The IOU threshold has been set to 0.7 after multiple trials which will obtain the best accuracy.

Furthermore, the authors have added attention mechanism after these 6 modifications. The residual connection in the original Yolo v3 has been replaced with Convolution Block Attention Module (CBAM) [36] to make the model pay more attention to the channels weights and able to focus on the important objects.

Although the Xie and Wei [11] method has performed a lot of modifications on the Yolo v3 model, the self-collected dataset by the method is quite small with only a total of 493 images. Therefore, their experimental result is less promising and the modifications might not be able to work well on other tasks. Although they use a small dataset, it is clearly noticeable that the attention mechanism does have better performance on the mAP metrics, from 0.5 to 0.95 with increments of 0.05 per step.

Suhr and Jung [13] proposed an end-to-end trainable one-stage parking slot detection model that extracts both the global and local parking slot information in a VGG16-based customized object detection model. The input image is fed through the VGG16 backbone to extract features and the obtained feature maps are further forwarded to a global and local information extractor. This method can identify the parking slot types, locate the parking slot position, and classify parking slot occupancy via the global and local information extractor. The global information extractor is responsible for obtaining the position, location, and occupancy of the parking slot and the local information extractor is responsible for obtaining the fine details of the parking slot which are the exact location of the junctions.

After obtaining both global and local information, the authors integrate this information to form the final parking slot output. A junction-based NMS is carried out during this integration, which the local junctions (fine output) will combine with the global junction (coarse) output when they are located near to each other, which is within 20 pixels, and the final parking slot can be determined. Since it is an end-to-end model, this method could be easily extended to other domains or datasets as no prior geometric knowledge is needed.

Wang et al. [14] proposed an end-to-end parking slot detection that utilized the Fully Convolutional One-Stage Object Detection (FCOS) [37] architecture. Instead of detecting the marking points of the parking slot, they took an approach detecting the four corners points instead. The feature pyramid network in FCOS is removed in this method as there will be no overlapping object in parking slot detection. Since this method is directly predicting the four corner points of a parking slot, it will eventually form a quadrilateral with the points given, yet, without a center as guidance, the model will tend to predict the corner points randomly. Therefore, the authors added in a centerness branch in the FCOS detection head. It will output a heatmap-like prediction result that shows the center of the parking slot and acts as guidance for the detection head that is responsible for predicting the four corner points. This method requires only one post-processing method, which is non maximum suppression. It is a simple and straightforward way to perform parking slot detection.

Bui and Suhr [18] proposed a one-stage parking slot detection method that consists of an information extractor and a post processing module called a progressive information assembly module. The information extractor consists of three main extractors: component extractor (Entrance Center [EC], junctions [JT], and separating line [SL]), linkage extractor (EC-JT Linkage, JT-SL Linkage), and property extractor (parking slot type and occupancy). These extractors will extract the information needed for the progressive information assembly module. The progressive assembly module will first connect the entrance center to the junctions using the vector obtained from the EC-JT linkage extractor. The precise location of the junctions will be calculated using the result from the junction extractor. The JT-SL linkage is then used to determine the orientation of the parking slot. Finally, by analyzing the cells inside the parking slot, parking slot type and occupancy could be determined.

#### 3.1.2. Multi-Stage Detection

Multi-stage detection methods in the context of parking slot detection involve multiple stages or post-processing steps to identify parking slots. These methods typically incorporate initial object or region proposals in the first stage, followed by subsequent stages that refine the analysis using techniques such as template matching or proposal classification. By progressively refining the detection process, multi-stage methods offer improved accuracy and robustness, but more computational resources must be invested.

Zhang et al. [4] proposed DeepPS which utilized Yolo v2 and a customized Deep Convolution Neural Network(DCNN) and was based on AlexNet to locate and classify the parking slot. In the first stage, with the use of a pre-trained Yolo v2 model, Zhang et al. fine-tuned the model to detect the marking point of the parking slot. After identifying the position of the marking points, the customized DCNN is used to classify these marking points into seven classes, which are:

1. right-angled anticlockwise;
2. slanted anticlockwise with an acute parking angle;
3. slanted anticlockwise with an obtuse parking angle;
4. right-angled clockwise;
5. slanted clockwise with an obtuse parking angle;
6. slanted clockwise with an acute parking angle;
7. invalid.

These classified marking points are then used to infer the parking slots with a “T-shaped” template matching strategy, where the distance between the marking points and the types of the marking point pairs are matched in order to identify whether the parking slot is slanted, parallel or perpendicular. This method is the first method that applies a deep learning strategy in parking slot detection.

Zinelli et al. [5] have used Faster R-CNN to conduct parking slot detection. Instead of a standard Faster R-CNN, an anchor-free based variant has been used due to the different possible shapes of the parking slot. First, the region proposal network (RPN) will generate a lot of proposals with an object score within the region. Next, at the detection head, the only region with an object score above 0.5 is kept and NMS is carried out to remove the excessive region proposal. The detection head will then classify the region whether it is background or an occupied or empty parking slot.

Wu et al. [8] used a hybrid approach of object detection and regression for the parking slot detection task. A circular descriptor is used in this method to have better robustness and generality in detecting various parking slot marking points. Since the marking points are relatively small in the image, this method splits the marking point detection and regression into two stages. In the first stage, the model will output a coarse region of the marking point and the region will be cropped out for the next stage. In the next stage, the sub-image will be fed into another CNN regression model to regress the exact location of the marking point. The circular descriptor employed in this approach proves effective in handling different types of marking points, such as trapezoid, brick, and oblique shapes. As the first stage operates similarly to a region proposal network, this method can be seen as a combined approach of object detection in the first stage and regression in the second stage.

Do and Choi [6] have proposed a context-based parking slot detector, with the use of a Mobile Net v2 as the context classifier and 3 Yolo v3 as the detector. In the first stage, the Mobile-Net v2-based classifier will identify whether the image has a slanted, perpendicular, parallel parking slot or no parking slot. If no parking slot is identified, the next stage will not be activated and vice versa, if one of the parking slot types is detected, its respective parking slot detector will be activated, therefore 3 Yolo v3 is required since there are three parking slot types. The detector is also responsible for detecting whether the parking slot is occupied. The rotated anchor boxes technique is used in this method to handle the different orientation of the parking slot too.

Li et al. [7] proposed VPS Net which is another two-stage parking slot detector where the first stage would be a Yolo v3 based detector and the second stage is a customized Alex Net to classify the occupancy of the detected parking slot. In the first stage, a pre-trained Yolo v3 is fine-tuned to train on the specific dataset (ps 2.0) to detect the marking points and parking slot head. The marking points detected in the bounding will be classified and inferred to identify the parking slot type, e.g., if an “obtuse-angled” marking point is identified in the bounding box, the parking slot will be a slanted parking slot. Then, with preset geometric cues, a complete parking slot could be inferred. In the next stage, the inferred parking slot will be regularized into small size and use to train the customized Alex Net classifier to classify parking slot occupancy.

Bui and Suhr [16] applied another region proposal network (RPN) based on DenseNet101 to execute parking slot detection. Instead of using the whole parking slot as the detection result, this method only utilizes the entrance of a parking slot, as the authors think parking slot entrance is sufficient to provide information. This method is a two-stage detection where the first stage uses the parking slot entrance to feed into the RPN and the second stage uses a detection network and classification network. In the first stage, the RPN will output a region of interest (ROI) that consists of the information of probability of entrance, location of entrance center, orientation of entrance, length of entrance, and orientation of parking slot. Using these obtained ROIs, the classification network will classify this information to realize the parking slot type and occupancy while the detection network will realize the precise location and orientation of the parking slot.

Chen et al. [10] used a multi-clue strategy to reconstruct and detect a parking slot. In the first stage, the input images are passed into an Illumination Balance Module to transform into gray images, then multiple image processing techniques are carried out: modified gaussian filter to reduce noise but not blur the image, area separation to extract the common area (parking slot) from the occulated view image, balance strategy to remove or balance the drastic change in illumination due to shadow.

Then, in the second stage, the corners (marking points) of the parking slot will be detected using spatial pyramid pooling techniques to improve the network learning ability and improve the local features. The third stage will be the multi-clue restoration strategy. Clues such as the sidelines, occlusion, edge, and domain are used to construct a complete parking slot.

**Figure 4:**
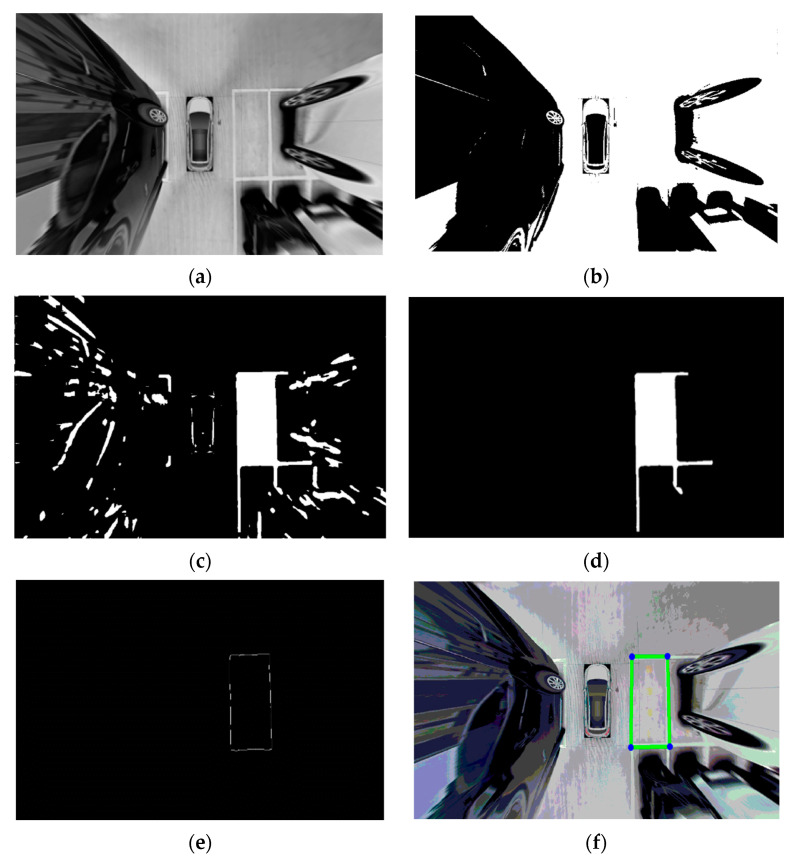
Results of the image processing techniques in order [9]: (**a**) Graying and image enhancement, (**b**) Binarization, (**c**) Extraction of connected region, (**d**) Corrosive operation, (**e**) Hough transform, and (**f**) Location coordinates transformation.

Huang et al. [12] used Faster R-CNN and image processing techniques for parking slot detection. This is a mixed approach of object detection and traditional computer vision techniques. In the first stage, the ResNet101-based Faster R-CNN network will output multiple regions of interest using the parking slot images. Then, the next stage will use these regions to carry out multiple image processing techniques to obtain a final binary edge image where the edge are the parking slot lines. The image processing techniques used are graying, image enhancement, binarization, extraction of connected region, corrosive operation, Hough transform, and location coordinates transformation. The results from these image processing techniques are shown in Figure 4.

Zheng et al. [15] proposed a Self-Calibration Convolution Network (SCCN) for parking slot detection. The SCCN backbone was the replacing of the ResNet-50 convolution layer with a self-calibration convolution layer. Firstly, the authors followed the ideas from CenterNet [38], where they computed a low-resolution feature map and predicted the center points of the entrance line using a heatmap. Next, MultiBin [39] architecture was used to estimate the directions of the entrance line and the separating lines. Lastly, grid cells were used to determine the area with a high probably of being occupied by vehicles. By using the entrance line obtained in the first stage and the orientation from the MultiBin, the authors were able to determine the parking slot type with predefined geometric rules.

Lee et al. [17] used CenterNet [38] as the parking slot detection baseline where they detected the location of the center of the parking slot, and the locations of the entrance points, parking slot type, and orientation. The method consisted of two detectors where a parking slot detector is used to predict the points and heatmap related to the center of the parking slot and a key point detector is used to predict the entrance points and its respective heatmap. After obtaining the center points and the key points, these points are associated to form the final parking slot.

### 3.2. Image Segmentation Approach

Image segmentation methods such as semantic segmentation or instance segmentation can be employed to accurately delineate the boundaries of parking slots and separate them from the background. Usually, methods that apply an image segmentation approach will segmentate the parking slot lines and include further post-processing techniques to identify a parking slot.

Wu et al. [19] used a High Fusion Convolutional Network (HFCN) with additional vertical and horizontal convolution kernel blocks, which they named the VH stage, to better differentiate and segmentate the lane markings and parking slot line. After obtaining the segmentation result of the lane marking and parking slot lines, three post processing steps were carried out to determine the final parking slot prediction result:

1. Morphological skeletonization is applied to extract the central path of each segmented area;
2. A Hough line transform on the skeleton is added to generate necessary lines;
3. Similar lines are partitioned and merged to build up the parking slots and lanes.

Jang and Sunwoo [20] proposed a custom encoder–decoder semantic segmentation network including the use of vertical grid techniques to generate a segmentation map of a parking slot image. Four classes will be segmentate via this network: the parking slot space, parking slot lines, vehicles, and other objects. After obtaining the segmentation map with each class being segmented, four steps will be taken: binarization, vertical grid encoding, grouping, and refinement. The segmentation map will be binarized in the form of a one vs. all technique and a vertical grid will be executed on each binarized map. The same class in a consecutive grid will be grouped together and the refinement process will be carried out to obtain the final segmentation result.

Jiang et al. [21] proposed DFNet which is adapted from PSPNet. It uses ResNet101 as the backbone/basic module, the pyramid pooling module from PSPNet as the feature extraction module, and a custom residual fusion block (RFB) for the refinement module. The RFB targets are used to refine the segmented area due to the noise generated during the up-sampling process. RFB is mainly focusing on the classification of the pixels at the boundary between two areas. To address the challenge of relatively small parking slot lines compared to the background, the authors of the study have implemented a weighting mechanism for the associated losses. Specifically, they have assigned weights to these losses based on the number of pixels classified to each class. In cases where more pixels are classified to a certain class, usually the background, the weight assigned to the loss of that class is reduced. This approach aims to mitigate the impact of the background class in the overall loss calculation, allowing the model to focus more on accurately detecting and segmenting the parking slot lines. By adjusting the weights, the authors aim to achieve a more balanced and effective training process that prioritizes the accurate detection and segmentation of the parking slot lines despite their relatively small size.

Yu et al. [24] specifically optimized the neural network via select and prune techniques in order to enable a real time performance. The backbone of the neural network is a stacked hourglass structure. This stacked hourglass structure enables salability in the task. The select and prune module is activated during the training process, where the least contributed convolution kernel will be selected and a pruned alternative. The contribution score of the kernels will be computed during the training process. The model will output a segmented map of the parking slot separation lines, the corners, and the entrance line. These segmented maps will be inferred and form the parking slots. The model size of this method is extremely small at only 2.39 MB.

Jian and Lin [22] cascaded 2 U-Net in order to separately detect the parking slot lines and corner points. The input image is fed into the first U-Net to produce a segmented map of parking slot entrance lines; this segmentation output is then fed into the next U-Net to obtain the segmented map of the corner points. These segmented entrance lines and corner point are further post-processed to filter out invalid corner points and locate the coordinates of a valid parking slot.

Jiang et al. [23] used Mask R-CNN with ResNet101 and FPN as the backbone to segment the marking points of a parking slot. In the first stage, the region proposal network will recommend the region that contains marking points, and the next stage will generate a mask that covers the marking points. The marking points mask is then used to detect lines using the line segment detection algorithm. Guidelines and parallel lines (separating lines) are used to identify the parking slot orientation.

**Figure 5:**
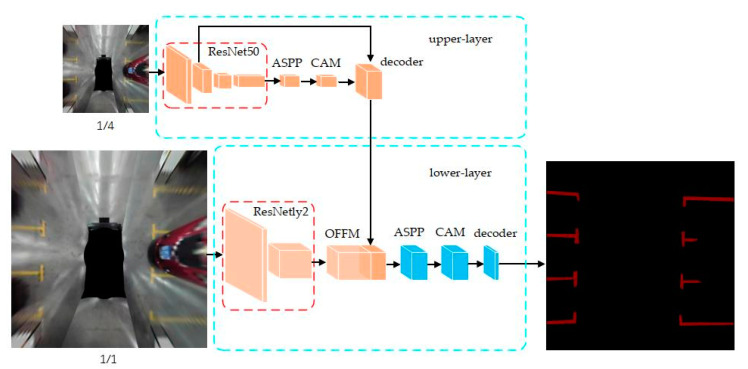
Double-layer segmentation network [25]. Upper layer output coarse segmentation result and pass to the lower layer for fine segmentation result.

Lai et al. [25] added a channel attention mechanism into the segmentation task of parking slot detection. The authors designed a double-layer segmentation network where the upper layer takes up ¼ size of the input image, resulting in an output of a coarse segmentation result for faster performance. This coarse segmentation result is cascaded into the lower segmentation network that takes a normal size input image and is responsible for a more detailed segmentation result. This double-layer network ensures a balanced speed and accuracy for real-time detection purposes. The channel attention mechanism is added to the decoding layer of both the upper and lower segmentation network. Dynamic weight loss is also used in this method to address the class imbalance issue between the background and parking slot lines. Figure 5 shows the double-layer network structure.

Lastly, Zhou et al. [26] added two attention-based blocks into the decoder to enhance spatial and channel correlation, namely the Position Attention Block (PAB) and the Multi-scale Fusion Attention Block (MFAB). This attention-based encoder–decoder network will output the segmentation of the parking slot lines, the corner points, and the entrance line. After obtaining the segmentation result, key points (the center points and the endpoints on both sides of the lines, and the center points of the corners) will be extracted from it. These key points are used in the instance matcher to predict the location and orientation of the parking slot.

### 3.3. Regression Approach

In the regression approach for parking slot detection, the focus lies in predicting the coordinates and orientation angle of the marking points within the parking slots. Once the coordinates of these marking points are obtained, template matching is applied, utilizing preset geometric rules, to infer the complete parking slot. By comparing the detected marking points with the predefined template, the system can determine the boundaries and shape of the parking slot.

Huang et al. [27] proposed a directional marking point regression (DMPR) with template matching to predict the parking slot. A custom CNN model is designed for this task and the output will be a 16 × 16 × 6 matrices, the 16 × 16 will be the grid for the input image and 6 will be the output value to be regressed, which are the (x, y) values of the marking point, the shape s, the cos θ and sin θ value of the marking point orientation, and the confidence score c. After obtaining the directional marking point, a template matching method is used to classify whether the marking point pairs are valid to form an entrance line. Thus, the corresponding parking c slot of the entrance line can be located.

Since DMPR is only able to predict perpendicular and parallel parking slots, Li et al. [28] proposed another parking slot detection method by treating this task as an entrance line regression problem. A custom CNN is designed for this entrance line regression and the output would be 16 × 16 × 9 matrices, just like in DMPR, predicting the entrance line coordinates point, angles, and confidence score. Since the entrance line coordinates are the ones that are being regressed, this method can predict slanted parking slots as well.

### 3.4. Graph Neural Network Approach

Graph Neural Network (GNN) is good for modeling global relations and has been applied to many computer vision tasks such a human pose estimation. Min et al. [29] were the first to apply GNN in the parking slot detection task. This method consists of three stages where, at the first stage, the image features are extracted via a feature extraction network. The extracted features are then fed to a marking point detector and marking point feature encoder. The detected marking points are passed to the position encoder to enhance feature representation and are fused with the marking point features.

In stage 2, the fused marking point features are then fed into an attentional graph neural network to aggregate the marking-point features. Multi-head attention is used in the graph neural network to improve expressivity.

In stage 3, the aggregated marking point features are then concatenated to form a 1 × 128 input feature for the entrance line discriminator, which is a simple feed forward network. The output of the entrance line discriminator is a K × 5 matrix, where K is the number of marking point pairs and 5 will be the coordination of the marking points and entrance line probability.

## 4. Discussion

**Figure 6:**
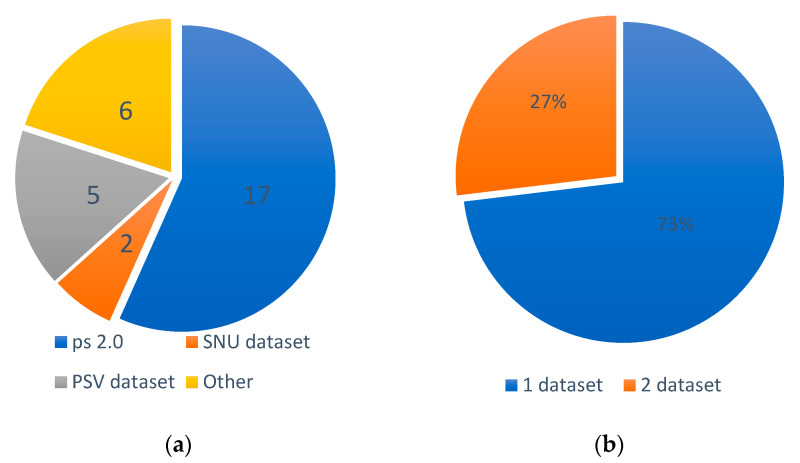
Dataset distribution, (**a**) dataset usage, (**b**) dataset usage per method.

In this section, the dataset and the parking slot detection method are summarized and discussed. The pie charts in Figure 6 show the distribution of the approaches as well as the usage of the dataset.

### 4.1. Dataset

Figure 6a illustrates the distribution of dataset usage among the reviewed works. It is observed that most of the works (17) opted to utilize the ps 2.0 dataset for evaluating their methods. Additionally, six of the works employed their own dataset (referred to as “other”) which was not publicly available. The SNU dataset, which was released in 2020, saw limited usage (two works) for method evaluation.

In Figure 6b, the evaluation strategy regarding the number of datasets used is depicted. Many works (73%) relied on a single dataset to assess their methods. However, 27% of the works employed two datasets in their evaluations. This involved either one public dataset combined with an additional self-labeled dataset or the utilization of two public datasets. The utilization of multiple datasets for evaluation purposes indicates a more promising approach, as it demonstrates the method’s ability to generalize effectively across different datasets. The details of each of these datasets is shown in Table 2 below.

#### 4.1.1. ps 2.0

**Figure 7:**
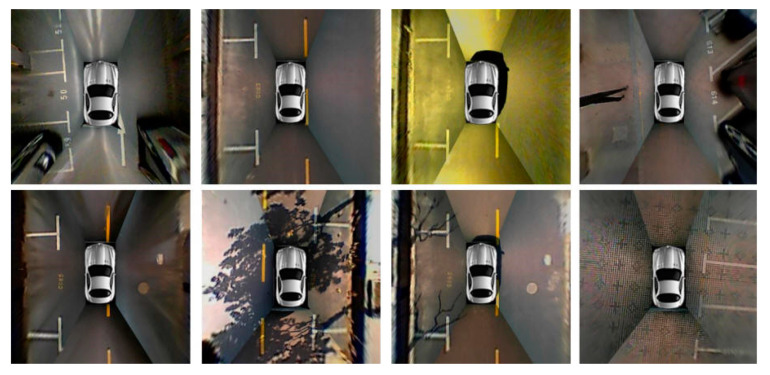
Image sample from ps 2.0 [4].

ps 2.0, introduced by Zhang et al. [4] from Tongji University, is a widely used dataset in the field of parking slot detection It comprises 9827 training images and 2338 test images, all captured at a resolution of 600 × 600 pixels (samples of the dataset are shown in Figure 7). The dataset was constructed by stitching together images obtained from four fisheye cameras, resulting in a bird’s-eye view representation. ps 2.0 encompasses a diverse range of parking lot environments, including both outdoor and indoor settings, and features three distinct parking lot types. Researchers have extensively utilized this dataset to evaluate and benchmark their parking slot detection methods, achieving remarkable levels of accuracy, with many studies reporting around 99% accuracy.

#### 4.1.2. PSV Dataset

**Figure 8:**
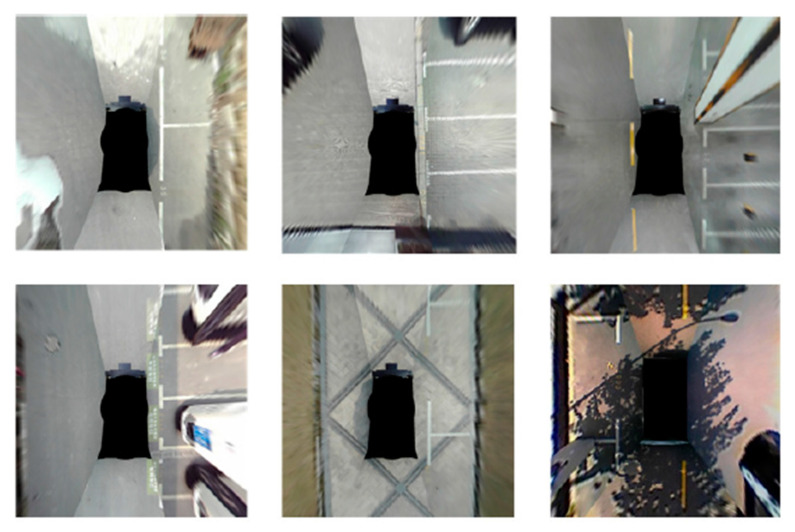
Image sample from PSV dataset [19].

The PSV dataset was introduced by Wu et al. [19] using TiEV (Tongji Intelligent Electronic Vehicle) based on roads in the Jiading campus of Tongji University. It comprises 2550 training images, 425 validation images, and 1274 test images, all captured at a resolution of 640 × 480 pixels. These dataset images were also captured on four fish-eye cameras and stitched together to form a bird-eye view image. Initially, this dataset was primarily intended for training image segmentation approaches. However, with the inclusion of additional annotation formats for object detection, it can also be utilized for training object detection models. Figure 8 provides the image samples from the PSV dataset.

#### 4.1.3. SNU Dataset

**Figure 9:**
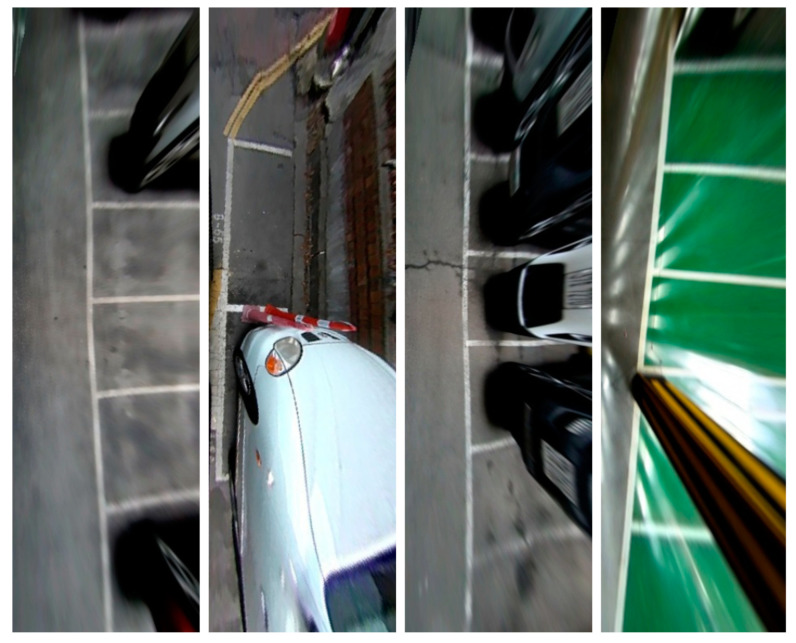
Image sample from SNU dataset [6].

The SNU dataset was released by Do and Choi [6] from Seoul National University. The SNU dataset consists of 18,299 images for training and 4518 images for testing of 768 × 256 pixels. The SNU dataset aims to serve as a superior replacement for ps 2.0 by offering more diverse scenes and various types of parking slots. To capture the dataset, fish-eye cameras were attached to the sides of the vehicle, and the images were not stitched together. Image samples are shown in Figure 9. This allows the detection algorithm to directly process each individual camera image without the need for stitching.

Although the SNU dataset has been released as a promising alternative to the ps 2.0 dataset, its utilization in research works for parking slot detection is still limited. The accuracy of methods evaluated using the SNU dataset has not reached an optimized stage compared to the ps 2.0 dataset. However, considering the dataset’s diversity and inclusion of different types of parking slots, it is highly encouraged that future research works utilize the SNU dataset. With further exploration and development, the SNU dataset holds great potential for advancing the accuracy and effectiveness of parking slot detection methods. Researchers are encouraged to leverage this dataset to enhance the performance and generalizability of their approaches in real-world parking scenarios.

### 4.2. Parking Slot Detection Methods Comparison and Discussion

Table 3, Table 4 and Table 5 provide a comprehensive summary of the different works conducted on various datasets. The results presented in the tables are obtained from the reported findings of each work. Works that only used self-label data and did not utilize any public datasets are not included in the tables as they are not comparable. The tables only include studies that conducted experiments using the standard test set, ensuring standardization and facilitating meaningful comparisons.

In line with the evaluation metrics proposed by Zhang et al. [4], the majority of the works assess their performance based on precision and recall rates. A detected parking slot is considered a true positive when the locations of its two junctions are within a threshold of 12 pixels from the ground truth, and their orientations are within 10 degrees of the ground truth. Precision is calculated as the ratio of true positives to the sum of true positives and false positives, while recall is calculated as the ratio of true positives to the sum of true positives and false negatives, as shown in Equations (1) and (2), respectively.

For image segmentation approaches utilizing the PSV dataset, an additional evaluation metric called mean Intersection over Union (mIOU) was employed to assess the quality of the segmentation results. The mIOU measures the overlap between the predicted segmentation mask and the ground truth mask for each class of objects in the image.

The detection speed, measured in frames per second (FPS), is also included. Considering that detection results may vary depending on the computational power of the hardware used, the hardware used by each work was also tabulated.

In the object detection approach, most of the works use either Yolo, Custom CNN, or Region Proposal Network (RPN) as their core architecture. These architectures are popular in the task of object detection as they provide good accuracy and detection speed. On the other hand, image segmentation tasks have utilized various architectures (Mask R-CNN, U-Net, etc.) to segment the parking slot lines. For a regression approach, custom CNN will be a better fit to generate the prediction result of marking points coordinates.

While most methods primarily focus on detecting different types of parking slots (slanted, perpendicular, parallel), it is worth noting that information about parking slot occupancy is crucial for autonomous driving. However, only 9 out of the 26 works included in the review incorporate parking slot occupancy as part of their detection results, emphasizing the importance of considering this aspect in future research endeavors.

**Figure 10:**
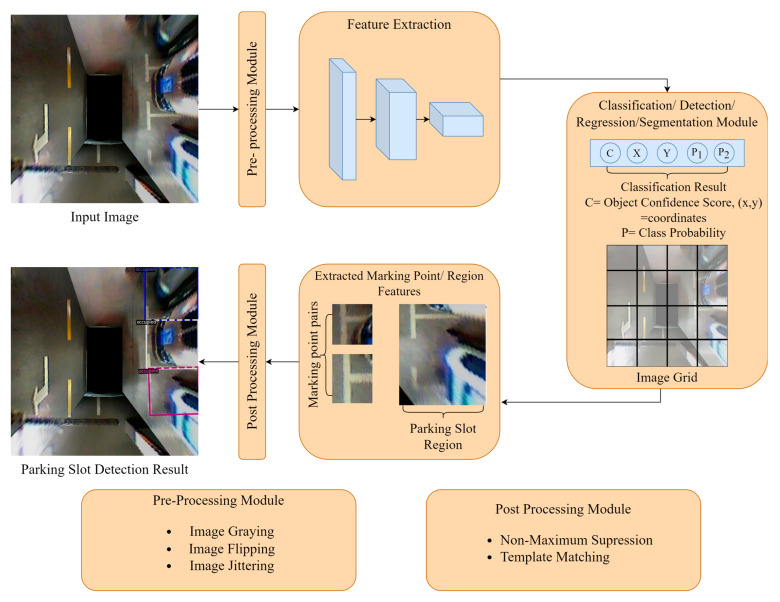
Summarized General Flow for Parking Slot Detection.

By summarizing these works, the parking slot detection flow could be generalized, as seen in Figure 10 below.

In the parking slot detection process, the input image is typically a surround view image generated by an Around View Monitor or a fisheye image captured by the fisheye camera. Before being fed into the feature extraction backbone, the input image undergoes preprocessing steps, such as resizing and data augmentation techniques like flipping or adding noise.

The feature extraction module plays a crucial role in extracting both low-level and high-level features from the parking slot. The low-level features can include corner points or color information that capture specific details of the parking slot. On the other hand, high-level features represent more abstract characteristics such as the overall shape, size, and arrangement of the parking slots.

These extracted features are then passed to the detection, classification, or segmentation module to generate predictions. The output predictions can take various forms, such as marking point coordinates, the delineated region of the parking slot, or a segmentation mask representing the boundaries of the parking slot.

After that, to refine the prediction output, post processing techniques are applied using predefined geometric rules or template matching. These rules help ensure that the predicted results align with the expected characteristics of parking slots.

## 5. Conclusions and Future Works

In conclusion, this review paper provides a comprehensive overview of vision-based deep learning approaches for parking slot detection on surround view images. The paper specifically focuses on deep learning methods and categorizes them into four main categories: object detection, image segmentation, regression, and graph neural network. Each category is thoroughly explained and reviewed, highlighting the key features and specialties of the methods.

Furthermore, the review analyzes three publicly available datasets: the ps 2.0, SNU dataset, and PSV datasets. The results obtained from works utilizing these datasets are presented, shedding light on the performance and advancements achieved in the field.

In addition to a comprehensive review of existing methods and datasets, this paper also identifies potential avenues for future research in vision-based deep learning parking slot detection on surround view images. Two key areas for future work are highlighted:

1. Establishing a more challenging and complete dataset.

The current publicly available datasets have nearly reached a bottleneck in terms of performance, especially ps 2.0, where most works utilizing this dataset have achieved 99% accuracy and above. There is a need to establish more diverse and comprehensive datasets that encompass a wider range of parking slot environments, including different lighting conditions, weather conditions, parking slot design variants, and more. In real world scenarios, there also exist parking slots that are unavailable for parking due to damaged or being occupied by some obstacle. These cases need to be taken into consideration too when collecting data for the dataset. Additionally, incorporating video-based data could be an important improvement to this research field, considering the real-time performance requirements of parking slot detection.

2. Optimizing the algorithm to run on an embedded system.

While the current research achieves fast and accurate results on powerful GPUs, it is important to consider the limitations of embedded systems in vehicles. Future research should focus on optimizing the detection algorithms to run efficiently on actual embedded systems, which may have lower computational capabilities. This can be achieved by exploring techniques to reduce the model’s FLOPS during inference or by leveraging embedded systems that are specifically designed for running deep learning models.

By examining a wide range of deep learning methods and evaluating them on various datasets, this review paper offers valuable insights into the current state of vision-based parking slot detection. It serves as a valuable resource for researchers and practitioners in the field, providing a comprehensive understanding of the different approaches and datasets available. The findings presented in this review can guide future research directions and advancements in vision-based parking slot detection using deep learning techniques.

## Figures and Tables

**Table 1:** Summary of research works on parking slot detection using deep learning approaches.

Literature	Year	Publication Source	Main Approaches
Zhang et al. [4]	2018	IEEE	Object Detection
Zinelli et al. [5]	2019	IEEE	
Do and Choi [6]	2020	IEEE	
Li et al. [7]	2020	MDPI	
Wu et al. [8]	2020	arXiv	
Xu and Hu [9]	2020	IOP	
Chen et al. [10]	2021	Springer	
Xie and Wei [11]	2021	IEEE	
Huang et al. [12]	2022	MDPI	
Suhr and Jung [13]	2022	IEEE	
Wang et al. [14]	2022	IEEE	
Zheng et al. [15]	2022	IEEE	
Bui and Suhr [16]	2023	IEEE	
Lee et al. [17]	2023	IEEE	
Bui and Suhr [18]	2023	IEEE	
Wu et al. [19]	2018	IEEE	Image Segmentation
Jang and Sunwoo [20]	2019	Springer	
Jiang et al. [21]	2019	IEEE	
Jian and Lin [22]	2020	IEEE	
Jiang et al. [23]	2020	MDPI	
Yu et al. [24]	2020	IEEE	
Lai et al. [25]	2022	MDPI	
Zhou et al. [26]	2022	IEEE	
Huang et al. [27]	2019	IEEE	Regression
Li et al. [28]	2020	Frontiers	
Min et al. [29]	2021	IEEE	Graph Neural Network

**Table 2:** Dataset breakdown.

Dataset	Resolution	Camera View	Training	Validation	Testing	Total
ps 2.0	600 × 600	Stitched Bird-eye view	9827	-	2338	12,165
PSV	640 × 480	Stitched Bird-eye view	2550	425	1274	4249
SNU	768 × 256	Vehicle side view	18,299	-	4518	22,817

**Table 3:** Comparison of parking slot detection methods using ps 2.0 dataset.

Approach	Literature	Vacancy Detection	Core Architecture	Precision	Recall	FPS	Dataset Split	Hardware
Object Detection	Zhang et al. [4]	No	Yolo v2	99.54%	98.89%	43	Default	Intel Xeon E5-2630V3 CPU, Nvidia Titan X
	Do and Choi [6]	Yes	MobileNet v2 + Yolo v3	98.70%	97.88%	23	Default	Intel i7-7700, GTX 1080
	Li et al. [7]	Yes	Yolo v3 + AlexNet	99.63%	99.10%	50	Default	Intel i9-7900X, 2× Nvidia Titan Xp
	Wu et al. [8]	No	Custom CNN	98.35%	99.60%	101	Default	Qualcomm 820a
	Chen et al. [10]	No	Custom CNN	93.21%	96.84%	-	Default	-
	Suhr and Jung [13]	Yes	VGG16 + Custom CNN	99.77%	99.77%	60	Default	GTX 1080 Ti
	Wang et al. [14]	No	FCOS	99.08%	99.40%	-	Default	-
	Zheng et al. [15]	Yes	SCCN	99.35%	99.17%	-	Default	RTX 2080 Ti
	Bui and Suhr [16]	Yes	Region Proposal Network (RPN)	99.77%	99.77%	45	Default	RTX 3090
	Lee et al. [17]	No	CenterNet	99.72%	99.63%	-	Default	-
	Bui and Suhr [18]	Yes	Custom CNN	99.63%	99.63%	134	Default	RTX 3090
Image Segmentation	Jian and Lin [22]	No	U-Net	99.40%	92.95%	-	Segmentation Labeled Test Set	-
	Yu et al. [24]	No	Stacked Hourglass	98.26%	97.56%	150	Segmentation Labeled Test Set	GPU (Not specified)
	Zhou et al. [26]	No	Attentional Encoder–Decoder Network	99.03%	98.57%	40	Segmentation Labeled Test Set	Nvidia P100
Regression	Huang et al. [27]	No	Custom CNN	99.42%	99.37%	83	Default	Nvidia Titan Xp
	Li et al. [28]	No	Custom CNN	99.68%	99.41%	77	Default	Intel i9-7900X, 2× Nvidia Titan Xp
Graph Neural Network	Min et al. [29]	No	VGG16 + GNN	99.56%	99.42%	40	Default	Nvidia Titan Xp

**Table 4:** Comparison of parking slot detection methods using PSV dataset.

Approach	Literature	Vacancy Detection	Core Architecture	Precision	Recall	mIOU	FPS	Dataset Split	Hardware
Object Detection	Li et al. [7]	Yes	Yolo v3 + AlexNet	96.54%	94.60%	-	50	Default	Intel i9-7900X, 2× Nvidia Titan Xp
	Suhr and Jung [13]	Yes	VGG16 + Custom CNN	96.33%	94.39%	-	60	Default	GTX 1080 Ti
Image Segmentation	Jiang et al. [23]	No	Mask R-CNN + Line Segment Detector	-	-	66.53	3	Default	Nvidia Titan X
	Wu et al. [19]	No	HFCN	-	-	46.51	9	Default	Nvidia Titan X
	Lai et al. [25]	No	ResNet50 + Attention Module	-	-	67.97	33	Default	Nvidia Titan Xp
Graph Neural Network	Min et al. [29]	No	VGG16 + GNN	97.05%	90.70%	-	40	Default	Nvidia Titan Xp

**Table 5:** Comparison of parking slot detection methods using SNU dataset.

Approach	Literature	Vacancy Detection	Core Architecture	Precision	Recall	FPS	Dataset Split	Hardware
Object Detection	Do and Choi [6]	Yes	MobileNet v2 + Yolo v3	87.75%	88.52%	23	Default	Intel i7-7700, GTX 1080
	Bui and Suhr [16]	Yes	Region Proposal Network (RPN)	95.78%	95.75%	45	Default	RTX 3090
	Bui and Suhr [19]	Yes	Custom CNN	96.75%	96.73%	134	Default	RTX 3090

## Data Availability

Not applicable.
